# Distal renal tubular acidosis presenting with an acute hypokalemic paralysis in an older child with severe vesicoureteral reflux and syringomyelia: a case report

**DOI:** 10.1186/s12882-022-02874-9

**Published:** 2022-07-14

**Authors:** Dara Ninggar Santoso, Fira Alyssa Gabriella Sinuraya, Cahyani Gita Ambarsari

**Affiliations:** 1 Department of Child Health, Faculty of Medicine Universitas Indonesia, Cipto Mangunkusumo Hospital Diponegoro, 71 Jakarta Pusat, 10430 Jakarta, Indonesia; 2 School of Medicine, University of Nottingham, Nottingham, UK; 3 Medical Technology Cluster, Indonesian Medical Education and Research Institute (IMERI), Faculty of Medicine Universitas Indonesia, Jakarta, Indonesia

**Keywords:** Anion gap, Chronic kidney diseases, Hydronephrosis, Intermittent urethral catheterization, Metabolic acidosis, Neurogenic urinary bladder, Spinal cord diseases, Urinary tract infections

## Abstract

**Background:**

Distal renal tubular acidosis (dRTA) is the most common type of renal tubular acidosis (RTA) in children. Pediatric dRTA is usually genetic and rarely occurs due to acquired issues such as obstructive uropathies, recurrent urinary tract infections (UTIs), and chronic kidney disease (CKD). Although persistent hypokalemia frequently occurs with dRTA, acute hypokalemic paralysis is not frequently reported, especially in older children.

**Case presentation:**

An eight-year-old girl presented with an acute first episode of paralysis. A physical examination revealed normal vital signs, short stature consistent with her genetic potential, and decreased muscle strength of her upper and lower extremities. Preexisting conditions included stage 4 CKD due to recurrent UTIs, severe vesicoureteral reflux and bilateral hydronephrosis, neurogenic bladder, and multisegment thoracic syringomyelia. Her laboratory work-up revealed hypokalemic, hyperchloremic metabolic acidosis with a normal anion gap. She also had a urine osmolal gap of 1.9 mOsmol/kg with a high urine pH. Intravenous potassium replacement resulted in a complete resolution of her paralysis. She was diagnosed with dRTA and discharged with oral bicarbonate and slow-release potassium supplementation.

**Conclusions:**

This case report highlights the importance of considering dRTA in the differential diagnosis of hypokalemic acute paralysis in children. Additionally, in children with neurogenic lower urinary tract dysfunction and recurrent UTIs, early diagnosis of spinal cord etiology is crucial to treat promptly, slow the progression of CKD, and prevent long-term complications such as RTA.

## Introduction

Renal tubular acidosis (RTA) is an inherited or acquired renal tubular defect that compromises the kidney’s ability to absorb filtered bicarbonate and excrete ammonia or titratable acid. The most common pediatric forms are distal (type 1) and proximal (type 2) RTA. Distal RTA (dRTA) is caused by impaired distal acid secretion that results in the inability to secrete hydrogen ions from the distal tubules [1].  The resulting clinical syndrome is characterized by hypokalemic, hyperchloremic metabolic acidosis with a normal anion gap, high urine pH (> 5.5), and in some cases nephrocalcinosis and nephrolithiasis [1–3]. 

Clinical manifestations of dRTA in children include polyuria, failure to thrive, constipation, Kussmaul breathing, and persistent hypokalemia [1–4].  This case report describes a late diagnosis of dRTA in a child previously diagnosed with stage 4 chronic kidney disease (CKD) due to neurogenic lower urinary tract dysfunction (NLUTD) secondary to syringomyelia. dRTA was first suspected when the patient presented with an acute episode of hypokalemic paralysis, a rare condition, particularly in older children.

## Case description

An eight-year-old girl was admitted to the emergency department with an acute primary episode of bilateral lower limb paralysis that had begun approximately 9 h prior. There was no history of trauma, loss of consciousness, rapid breathing, fever, seizure, spasm, gastrointestinal tract losses, polyuria, paresthesia, or pain. She had a normal developmental history, normal auditory function, and experienced no leg weakness or sensory loss previously. No family members reported similar medical history or kidney diseases, and her parents were not close relatives. Her physical examination revealed that she was fully alert with normal vital signs. She was well nourished with short stature (height < 3rd percentile) consistent with her genetic potential. She had normal physiological reflexes. Muscle strength was 4/5 for the upper limbs and 3/5 for the lower limbs. No pathological reflexes, clonus, spasticity, or rigidity was found.

**Fig. 1:**
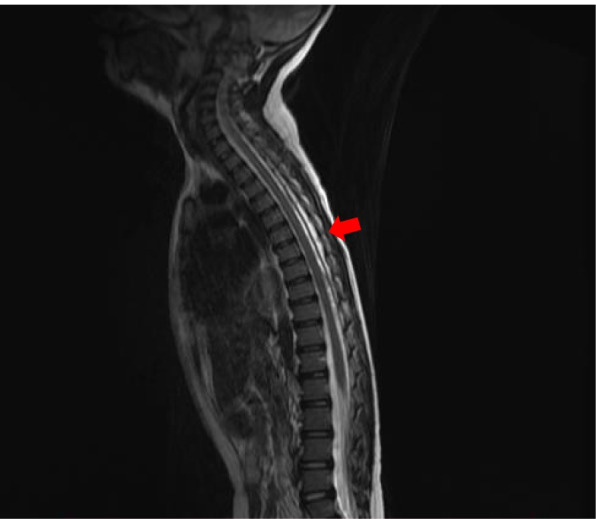
Sagittal T2-weighted whole spine magnetic resonance imagings (MRIs) of the patient indicating syringomyelia at T2–T7 level (arrow)

The patient had a history of anal atresia with a rectovaginal fistula that was fully corrected when she was 1 year old. She had recurrent urinary tract infections (UTIs) caused by high-grade vesicoureteral reflux (VUR) and severe bilateral hydronephrosis. She was treated with prophylactic antibiotics. When she was 6 years old, a bulking agent was used to treat bilateral VUR. She also received tamsulosin (α-1 blocker) at the age of 6 years which was then discontinued after 3 months due to no improvements. Urodynamics were performed and suggested bladder outlet obstruction with a residual urine volume of 150 mL. Magnetic resonance imaging (MRI) of the spinal cord revealed syringomyelia extending from thoracic spine Th2 to Th7 (Fig. 1). Clean intermittent catheterization (CIC) was initiated and maintained until admission. No breakthrough UTIs were noted. After a 2-year follow-up, the hydronephrosis persisted.

Initial laboratory investigations revealed a normal complete blood count and electrolyte imbalances, notably hypokalemia (K 2.4 mEq/L). Urinalysis revealed a UTI (Table 1). The electrocardiogram (ECG) indicated a normal heart rate and T wave inversions. The patient had decreased kidney function corresponding to an estimated glomerular filtration rate (eGFR) of 25 mL/min per 1.73 m^2^  (Table 1). She was treated with intravenous (IV) potassium chloride, sodium chloride, and cefotaxime and investigated for possible etiologies of hypokalemic paralysis. On the third day of hospitalization, her venous blood gasses and electrolytes indicated hyperchloremic metabolic acidosis with hypokalemia and a normal anion gap. The urine pH was 6.5 with a positive urine anion gap. Her urinary pH before this admission has ranged from 7.0 to 7.5. The urinary calcium creatinine ratio was 0.19 mg/g. Thyroid function was within the normal range. Urine osmolal gap (UOG) when she was free from UTI was 1.9 mOsmol/kg (Table 2). Kidney ultrasonography (US) did not document any nephrocalcinosis.

**Table 1:** Laboratory investigation on admission

Parameter	Unit	Result	Reference range
**Serum electrolytes**			
Sodium	mEq/L	**128**	135–145
Potassium	mEq/L	**2.4**	3.5–4.5
Chloride	mEq/L	**115**	98–107
Calcium (ionized)	mmol/L	1.09	1.01–1.31
**Kidney function**			
Blood urea nitrogen	mg/dL	**20.5**	7–16.8
Serum creatinine	mg/dL	**1.8**	0.3–0.6
Estimated glomerular filtration rate^a^	mL/min per 1.73 m ^2^	**25**	89–165
**Urinalysis**			
Color		Yellow	
Clarity		Cloudy	Clear
pH		6.5	4.5–8
Specific gravity		< 1.005	1.005–1.030
Glucose		Negative	Negative
Blood		**1+**	Negative
Ketones		Negative	Negative
Protein		**1+**	Negative
Urobilinogen		3.2	3.2–16
Bilirubin		Negative	Negative
Leukocyte esterase		**2+**	Negative
Nitrite		**Positive**	Negative
**Urine culture**			
Microorganism	colony-forming unit /mL	**Escherichia coli > 100,000**	No growth

^a^serum creatinine-based eGFR (Schwartz formula-calculated)

**Table 2:** Subsequent laboratory investigation

Parameter	Unit	Result		Reference Range
		3rd day of hospitalization	After the UTI^a^ resolved	
**Serum electrolytes**				
Sodium	mEq/L	138		135–145
Potassium	mEq/L	3.2		3.5–4.5
Chloride	mEq/L	115.9		98–107
Calcium (ionized)	mmol/L	1.1		1.01–1.31
Phosphate	mg/dL	4.6		4–7
Magnesium	mg/dL	1.76		1.7–2.1
**Venous blood gas analysis**				
pH		**7.256**		7.35–7.45
pCO_2_	mmHg	**30.2**		35–45
pO_2_	mmHg	22.1		75–100
HCO_3_	mmol/L	**13.6**		21–25
Base excess	mmol/L	**-11.6**		-2.5 – +2.5
O_2_ saturation	%	49.1		60–80
**Urinalysis**				
Color		Yellow	Yellow	Clear
Clarity		Clear	Clear	4.5–8
pH		7.5	7.5	1.005–1.030
Specific gravity		< 1.005	< 1.005	Negative
Glucose		Negative	Negative	Negative
Blood		**1+**	**Trace**	Negative
Ketones		Negative	Negative	Negative
Protein		**Trace**	Negative	3.2–16
Urobilinogen		3.2	3.2	Negative
Bilirubin		Negative	Negative	Negative
Leukocyte esterase		**Trace**	Negative	Negative
Nitrite		Negative	Negative	Clear
**Urine electrolytes and metabolites**				
Sodium	mEq/L	25	31	(> 20)
Potassium	mEq/L	**11.3**	9.4	20–80
Chloride	mEq/L	**34**	29	46–168
Urine anion gap	mEq/L	2.3	11.4	0–10
Urea nitrogen	mmol/L		53.6	N/A
Glucose	mmol/L		0	0–0.8
Calculated urine osmolality	mOsmol/kg		134.4	
Measured urine osmolality	mOsmol/kg		136.3	400–800
Urine osmolal gap	mOsmol/kg		1.9	10–100
Calcium creatinine urine ratio	mg/g	0.19		< 0.2
**Thyroid function**				
fT4	ng/dL	1.04		0.89–1.37
TSHs	µIU/mL	4.18		0.35–4.94

^a^*UTI* urinary tract infection

dRTA was therefore diagnosed. The paralysis resolved completely, the urinalysis normalized, and the patient was discharged with oral bicarbonate 500 mg every 8 h and potassium slow release (KSR) 1200 mg every 12 h.

## Discussion

Our patient was hospitalized after her first episode of acute hypokalemic paralysis. Based on her history and physical examination, we concluded that she had a lower motor neuron type paralysis originating from the muscles along with hypokalemia (K 2.4 mEq/L), suggesting an acute hypokalemic paralysis. The possible cause of hypokalemia was investigated, primarily the possibility of potassium loss from the kidneys. The occurrence of hypokalemia and metabolic acidosis suggests the possibility of a gastrointestinal loss of bicarbonate or RTA [5].  In this case, RTA was considered the possible cause due to the patient’s obstructive uropathy and the absence of gastrointestinal loss. All forms of RTA induce normal anion gap (hyperchloremic) metabolic acidosis, but the hallmark of dRTA is impaired urine acidification [5–7].  The findings of hyperchloremic metabolic acidosis, a normal anion gap, a positive UAG, a UOG of < 150 mOsmol/kg, and a high urine acidity (pH > 5.5) support the diagnosis of dRTA. Urine anion gap and UOG are indirect markers of urine acidification that estimate urinary ammonium (NH_4_^+^), which represents a major portion of net acid excretion (NAE) [6, 7].  The urine acidification impairment in dRTA and CKD patients is associated with positive UAG and a decrease in UOG, with both markers are well correlated with urine NH_4_^+^ and NAE [6, 7]. However, UOG provides a better estimation of urinary NH_4_^+^ excretion than UAG in RTA, due to the latter becoming a less reliable predictor of urinary NH_4_^+^ excretion in patients with acute or chronic kidney disease [8, 9].

Hypokalemic paralysis is a relatively uncommon clinical manifestation of hypokalemia in patients with dRTA, and usually presents in cases of severe hypokalemia [2–4, 10]. In this case, however, hypokalemic paralysis occurred with a moderate level of hypokalemia. This may be due to chronic hypokalemia that was late to be discovered. In our setting, the limited availability of supporting diagnostic tools and laboratories has been a major constraint that has frequently caused diagnosis and treatment delays, particularly in patients living in remote areas, such as our current case [11, 12] .

Pediatric dRTA can be genetic or acquired. Genetic causes are most often reported in pediatric cases, although the condition can also occur due to drugs and toxins (e.g., amphotericin B and lithium), obstructive uropathies, pyelonephritis, interstitial nephritis, CKD of any cause, or autoimmune diseases (e.g., Sjögren syndrome and systemic lupus erythematosus) [1, 3]. The clinical manifestations of dRTA are further classified based on the underlying etiology. The recessive genetic form presents in infancy and is associated with more severe symptoms, while the dominant form presents later in life as a milder disease [1, 2].  Acquired dRTA may occur at any age, depending on the timing of the tubular injury of the kidneys [1].

Patients with the recessive genetic form of dRTA may present with severe hyperchloremic metabolic acidosis, moderate to severe hypokalemia, nephrocalcinosis, vomiting, dehydration, poor growth, rickets disease, and/or bilateral sensorineural hearing loss [1–4].  In comparison, the dominant genetic form is usually associated with milder disease: the most frequent initial finding is kidney stones or nephrocalcinosis, and patients typically have mild metabolic acidosis and mild to moderate hypokalemia [1, 2]. The underlying pathophysiology of dRTA consisted of decreased net activity of proton pumps and increased luminal membrane hydrogen ion permeability [5, 6]. The secretion of H^+^ into the lumen of the distal tubule is accomplished by type A intercalated cells of tubules via H-ATPase and H-K-ATPase pumps, which secrete H^+^ and reabsorb potassium [13]. Defects in these proton pumps reduce H + secretion and increase net potassium excretion by decreasing potassium reabsorption, causing potassium wasting and subsequently, hypokalemia [5, 6]. Hypokalemic paralysis usually presents in those with moderate to severe hypokalemia (K < 2.5 mEq/L).^4^ The defective H-ATPase also generates sodium wasting [14]. In addition, the state of metabolic acidosis may inhibit a component of the proximal tubule for sodium reabsorption [15]. The luminal membrane of the distal nephron is required to be relatively permeable to H + and carbonic acid to generate and maintain highly acidic urine. In dRTA, there is an increased permeability that results in back-leak of secreted H + from the urine into the extracellular fluid, which reduces urine acid excretion [9]. Therefore, the initial findings of hyperchloremic metabolic acidosis, hypokalemia, and hyponatremia in our patient.

The decrease in kidney function in our patient was a consequence of recurrent complicated UTIs due to severe bilateral VUR and hydronephrosis due to chronic NLUTD. The syringomyelia on the T2–T7 vertebrae segment is linked as the underlying etiology for her NLUTD. Recurrent UTIs in children with structural kidney and urinary tract abnormalities are a major risk for CKD [16]. NLUTD and obstructive uropathy have also been reported as significant causes of end-stage kidney disease in children, including in our country [17, 18].

Cases of syringomyelia presenting with neurogenic bladder have rarely been reported. Reports of dRTA secondary to obstructive uropathies due to spinal cord diseases are particularly rare [19].  dRTA in children is typically a primary genetic defect. However, we consider our case to be acquired dRTA due to the obstructive uropathies and history of recurrent UTIs. Genetic testing was not performed because the clinical history completely explained her clinical presentation. Additionally, the patient lacked any hearing impairment, nephrocalcinosis, or rickets, which are hallmarks of the genetic forms of dRTA.

Our patient was discharged after a complete resolution of symptoms due to potassium administration. She is being maintained on lifelong supportive treatments with regular oral potassium and bicarbonate supplementations. Other urinary alkalinizer such as sodium citrate (Shohl’s solution) and potassium citrate are commonly used for the treatment of dRTA as they have an additional benefit to reduce the risk of urinary tract stones formation [20, 21]. However, Shohl’s solution is not available in Indonesia, whereas potassium citrate is exclusively available only in our center with an inconsistent supply. Therefore, our case was discharged with oral bicarbonate and KSR. The parents were informed about the general management, the importance of adherence to long-term treatment, and possible poor prognosis of stage 4 CKD. Metabolic profiles and kidney function need to be monitored regularly to prevent recurrence of symptoms, future nephrocalcinosis, bone deformities, and growth retardation which may occur in the untreated dRTA [22].

This case report highlights the importance of considering dRTA in the differential diagnosis of hypokalemic acute paralysis in children. Additionally, in children with neurogenic lower urinary tract dysfunction and recurrent UTIs, early diagnosis of spinal cord etiology is crucial to treat promptly, slow the progression of CKD, and prevent long-term complications such as RTA.

## Abbreviations

CIC: Clean intermittent catheterization
CKD: Chronic kidney disease
dRTA: Distal renal tubular acidosis
ECG: Electrocardiogram
eGFR: Estimated glomerular filtration rate
IV: Intravenous
MRI: Magnetic resonance imaging
NAE: net acid excretion
NLUTD: Neurogenic lower urinary tract dysfunction
RTA: Renal tubular acidosis
UAG: Urine anion gap
UOG: Urine osmolal gap
US: Ultrasonography
UTIs: Urinary tract infections
VUR: Vesicoureteral reflux
